# An Attention towards the Prophylactic and Therapeutic Options of Phytochemicals for SARS-CoV-2: A Molecular Insight

**DOI:** 10.3390/molecules28020795

**Published:** 2023-01-13

**Authors:** Shoaib Shoaib, Mohammad Azam Ansari, Geetha Kandasamy, Rajalakshimi Vasudevan, Umme Hani, Waseem Chauhan, Maryam S. Alhumaidi, Khadijah A. Altammar, Sarfuddin Azmi, Wasim Ahmad, Shadma Wahab, Najmul Islam

**Affiliations:** 1 Department Biochemistry, Faculty of Medicine, J. N. Medical College, Aligarh Muslim University, Aligarh 202002, India; 2 Department of Epidemic Disease Research, Institute for Research and Medical Consultations (IRMC), Imam Abdulrahman Bin Faisal University, Dammam 31441, Saudi Arabia; 3 Department of Clinical Pharmacy, College of Pharmacy, King Khalid University (KKU), Abha 62529, Saudi Arabia; 4 Department of Pharmacology, College of Pharmacy, King Khalid University (KKU), Abha 62529, Saudi Arabia; 5 Department of Pharmaceutics, College of Pharmacy, King Khalid University (KKU), Abha 62529, Saudi Arabia; 6 Department of Zoology, Faculty of Life Sciences, Aligarh Muslim University, Aligarh 202002, India; 7 Department of Biology, College of Science, University of Hafr Al Batin, Hafr Al Batin 31991, Saudi Arabia; 8 Molecular Microbiology Biology Division, Scientific Research Centre (SRC), Prince Sultan Military Medical City (PSMMC), Riyadh 11159, Saudi Arabia; 9 Department of Pharmacy, Mohammed Al-Mana College for Medical Sciences, Dammam 34222, Saudi Arabia; 10 Deparment of Pharmacognosy, College of Pharmacy, King Khalid University, Abha 61421, Saudi Arabia

**Keywords:** phytochemicals, polyphenols, SARS-CoV-2, antiviral, therapeutics, anti-inflammation, antioxidant, prevention

## Abstract

The novel pathogenic virus was discovered in Wuhan, China (December 2019), and quickly spread throughout the world. Further analysis revealed that the pathogenic strain of virus was corona but it was distinct from other coronavirus strains, and thus it was renamed 2019-nCoV or SARS-CoV-2. This coronavirus shares many characteristics with other coronaviruses, including SARS-CoV and MERS-CoV. The clinical manifestations raised in the form of a cytokine storm trigger a complicated spectrum of pathophysiological changes that include cardiovascular, kidney, and liver problems. The lack of an effective treatment strategy has imposed a health and socio-economic burden. Even though the mortality rate of patients with this disease is lower, since it is judged to be the most contagious, it is considered more lethal. Globally, the researchers are continuously engaged to develop and identify possible preventive and therapeutic regimens for the management of disease. Notably, to combat SARS-CoV-2, various vaccine types have been developed and are currently being tested in clinical trials; these have also been used as a health emergency during a pandemic. Despite this, many old antiviral and other drugs (such as chloroquine/hydroxychloroquine, corticosteroids, and so on) are still used in various countries as emergency medicine. Plant-based products have been reported to be safe as alternative options for several infectious and non-infectious diseases, as many of them showed chemopreventive and chemotherapeutic effects in the case of tuberculosis, cancer, malaria, diabetes, cardiac problems, and others. Therefore, plant-derived products may play crucial roles in improving health for a variety of ailments by providing a variety of effective cures. Due to current therapeutic repurposing efforts against this newly discovered virus, we attempted to outline many plant-based compounds in this review to aid in the fight against SARS-CoV-2.

## 1. Introduction

In December 2019, a novel pathogenic strain of virus emerged in Wuhan, China that has since spread all over the world within a short period of time [1]. Further examination of isolated sputum samples revealed that the virus is etiologically distinct from other coronaviruses known as the 2019 novel coronavirus (2019-nCoV) or severe acute respiratory syndrome (SARS) coronavirus 2 (CoV2) [2], both of which belong to the Coronaviridae family. With its rates of high transmissibility and exacerbating mortality, the virus imposed a global emergency and the threat of more deaths. This coronavirus resembles most of the characteristics of other coronaviruses such as SARS-CoV and MERS-CoV. Worldwide, scientists are emphasizing the need to understand its rapid transmissibility, genetic proximity, and severity as the pandemic appears increasingly oppressive to humans [3]. The data indicated its transmission occurs mainly through the droplets of an infected individual, but sometimes it could also follow a nosocomial mode of transmission. Meanwhile, many researchers disclosed the viral structure, which consists of an enveloped non-segmented positive-sense RNA, nucleocapsid protein, and spike glycoprotein, in addition to other structural membrane proteins. The transmission implies viral entry through the binding of the spike protein to the cellular receptor present on host cell surfaces, which is further identified as angiotensin-converting enzyme 2 [4,5]. Additionally, for a better understanding, the viral genome has been sequenced to enable the diagnosis, origin tracking, and the development of therapeutics and preventive strategies. So far, the most acceptable method available for the diagnosis of SARS-CoV-2 is PCR-based [6]. The severity of the disease may depend upon a number of factors such as age, gender, and the presence of secondary infections, poor immunity, and the level of inflammatory cytokines in the blood [7]. The majority of SARS-CoV-2-infected patients presented mild flu-like symptoms such as a fever and a cough [8]. Patients infected with SARS-CoV-2 may develop symptoms such as sputum production, headache, hemoptysis, diarrhea, dyspnea, and lymphopenia, according to several other clinical studies [9,10,11]. However, the critical patients rapidly developed acute respiratory distress syndrome (ARDS), hypertension, cardiovascular disease, kidney disease, and other associated problems [12]. Another recent clinical finding showed gastrointestinal problems and asymptomatic infections, especially in children [13]. People with a poor immune system, inflammatory disease, and those with renal and hepatic dysfunction were indicated to be at greatest risk of SARS-CoV-2 infection [14]. Symptomatic and asymptomatic evidence suggests the severity of the disease, which could possibly be acting as a transmission factor, thus continuing to spread all over the world. To date, several vaccines and drug candidates have been developed, and many are in clinical trials; yet, there is an unmet demand for an effective and target-specific drug regimen with fewer side effects. Therefore, the current situation urges researchers to design more therapeutic strategies with high efficacy but low adverse side effects, which will minimize viral load and prevent SARS-CoV-2 infection.

## 2. Are Immunological and Pathophysiological Findings Helping in the Treatment, Prevention and Control of SARS-CoV-2?

Severe acute respiratory syndrome-coronavirus 2 (SARS-CoV-2) has recently been shown to interact with the renin-angiotensin-aldosterone system through ACE2, which is known as the SARS-CoV cell receptor in humans with 10- to 20-fold higher affinity, which could be responsible for its rate of higher transmission [15]. Currently, the majority of clinical data show that SARS-CoV-2 uses the same ACE2 receptor, as confirmed by the analysis of patient bronchoalveolar lavage [16]. The virus has several proteins with various functions, but the spike protein is essential for attaching to the receptor (ACE2) that is found to be available on the surface of human cells. ACE2 is a member of the dipeptidyl carboxydipeptidases group and may be a potential infectivity factor due to its high interaction with SARS-CoV-2 [17,18]. As a result of the binding of SARS-CoV-2 with ACE2, proteolytic cleavage of the receptor occurs, leading to viral invasion and entry into the surrounding cells, which is facilitated by a transmembrane serine protease 2 [15]. Moreover, the clinical findings of most of the patients have shown elevated levels of pro-inflammatory cytokines, an abnormal respiratory rate, and an increased number of leukocytes. Furthermore, the number of neutrophils, D-Dimer, blood urea, and creatinine levels were also considerably higher in critically ill patients [19]. Several other studies have proven abnormal pathological findings for COVID-19 patients, which include a high erythrocyte sedimentation rate (ESR) and an increased level of C-reactive protein (CRP), suggesting significantly higher levels of inflammatory cytokines in these patients [6,20]. The cytokines and chemokines were significantly high in the blood of COVID-19 patients. Another study reported that critically ill patients exhibited a lower lymphocyte count and oxygen saturation, whereas higher controlled values were observed for white blood cell count, CRP, ferritin, and lactate dehydrogenase [21]. Excessive levels of cytokines in the blood resulted in the activation of more immune cells, causing a complication that is called a “cytokine storm” [22]. A cytokine storm resembling disturbed cytokine profile is strongly associated with the disease severity of COVID-19 patients, which may be characterized by significantly high levels of interleukin (IL)1-β, IL7, IL8, IL9, IL10, fibroblast growth factor (FGF)-2, granulocyte colony-stimulating factor (G-CSF), granulocyte-macrophage colony-stimulating factor (GM-CSF), interferon (IFN)-γ, Monocyte chemoattractant protein-1 (MCP1), MIP1α, MIP1β, and tumor necrosis factor (TNF)-α [12]. In a multicenter cohort study, the critically ill patients with SARS-CoV-2 were also diagnosed with lymphocytopenia along with increased levels of IL-6 and lactate dehydrogenase, which were indicated to be the prominent immunopathological markers for SARS-CoV-2 diagnosis [23,24,25]. Many other immunological findings suggest a reduced number of T cells and NK cells in COVID-19 patients while critically ill. COVID-19 patients have very low or even undetectable levels of NK cells. The most probable rationale driving the correlation between disease progression and reduced levels of NK cells may be functional exhaustion of cytotoxic lymphocytes [26].

Another study reported that the overall NK and CD8^+^ T cell count decreased significantly with increased expression of natural killer cell receptor G2A (NKG2A) in COVID-19 patients. Particularly, the number of NK and CD8^+^ T cells had been restored to reduce the expression of NKG2A in patients recovering after treatment. These results indicate that the functional exhaustion of antiviral lymphocytes is associated with SARS-CoV-2 [27]. A recent study showed that the majority of critically ill patients admitted showed signs of organ function damage, including acute respiratory distress syndrome (ARDS) and injuries in the kidney and heart [28]. In another study, out of 138 COVID-19 patients, 97 were reported to have lymphopenia, prolonged prothrombin time (PTT) was found in 80 patients, and an elevated level of lactate dehydrogenase in 55 patients, which could impose several other serious pathophysiological conditions. Further investigations showed that 36 patients who were admitted to the ICU showed ARDS, dyspnea, arrhythmia, and shock, and 64 patients were found to have one or more comorbidities, including with hypertension, diabetes, cardiovascular disease, and malignant neoplasms [29]. In a recent study, it was noted that critically ill patients admitted to the ICU were persisting with several comorbidities, including hypertension, acute hypoxemic respiratory problems, chronic obstructive pulmonary disease (COPD), cardiovascular problems, hypercholesterolemia, chronic kidney and liver diseases, and type 2 diabetes. Most of these patients were admitted due to hypertension and acute hypoxemic respiratory problems [30]. The pathophysiological findings such as cell degeneration, necrosis, macrophage proliferation, and phagocytosis of the spleen, and destroyed secondary lymphoid tissues were recently documented, which is a very striking aspect of COVID-19 patients [31]. Identifying and treating the cytokine storm with existing therapeutics with proven safety and low side effects may help to meet the immediate need for reducing the mortality rate and preventing rapid transmission.

## 3. Is the Drug-Repurposing Approach Sufficient? Or Novel Drug Development Is the Need to Curb SARS-CoV-2

Up to now, several drug candidates for SARS-CoV-2 have been repurposed worldwide, and ongoing research also focuses on the identification and development of new drug candidates (natural or synthetic). Use of SARS-CoV-2 disinfectants is one of the most well-recognized preventive measures against this virus; however, SARS-CoV-2 disinfectants have also imposed environmental and health impacts, including the formation of poisonous and mutagenic by-products due to disinfectant-based pollution of water bodies, air, and soil [32]. To date, the drugs being studied include revamped influenza medications, anti-malarial drugs, ineffective Ebola drugs, as well as SARS-CoV and MERS-CoV, which were initially developed decades ago. Various approaches have been put forward for the treatment of coronavirus to curb the viral load, and some of the older antiviral, anti-malarial, and other drugs can be correlated with the positive outcomes against coronavirus, but much more efforts are needed to fight SARS-CoV-2. Almost three years have passed since the SARS-CoV-2 pandemic began, but it remains uncertain which drugs or drug combinations could efficiently combat the viral infection. However, during this period, drug repurposing, as well as the identification of plant-based products, remained of keen interest among researchers worldwide. While developing a more effective vaccine takes time due to clinical trials, the world hopes to prevent the spread of the novel coronavirus soon with ongoing, intensive efforts.

Although there were initially no FDA-approved therapeutics for the treatment and prevention of SARS-CoV-2, several FDA-approved therapies are now being used to alleviate symptoms from a supportive care standpoint [33]. Still, the best solution to combat the recurrent spread of SARS-CoV-2 is to test whether the proven antiviral drugs have the ability to cure this viral infection. As of now, several possible predictions, including key regulatory molecules participating in the central dogma of molecular biology, have been envisaged and accepted as molecular targets. Selectively, monoclonal antibodies, peptides, interferons, oligonucleotides, and plant products have been tested in search of potential chemopreventive and chemotherapeutic drug candidates for the prevention and treatment of the newly emerging coronavirus. Here we have discussed many of the drugs for which doctors around the world are hoping to find a treatment to combat and overcome the spread of SARS-CoV-2. This review includes antiviral plant-based compounds against various molecular targets, including ACE2 and transmembrane serine protease 2 (TMPRSS2), RNA-dependent RNA polymerase (RdRp), and 3C-like cysteine protease (3CL pro), targeting inhibition of membrane fusion, assembly, and replication.

Recently, some of the small molecules, including remdesivir, galidesivir, ribavirin, and favipiravir, have been considered potent inhibitors of the enzyme RNA-dependent RNA polymerase, which possesses a binding pocket to interact with these chemical structures [34]. The outbreak, however, has addressed the urgent need for sustained efforts to develop broad-spectrum antiviral drugs to fight against such a virus with high morbidity. It has been validated in several research studies that viral replication is targeted by these nucleoside analogues to hamper DNA or RNA polymerase activity [35]. Many of the nucleoside analogs tested previously have low efficacy, as well as an inability to prevent coronavirus replication, and thus low selectivity to interfere with virus growth, whereas galidesivir is a nucleoside analogue that inhibits coronavirus replication [36]. Remdesivir is a nucleoside analogue (GS-5734) inhibiting viral perpetuation through targeting the two key enzymes (polymerase and exoribonuclease) [37] and pre-mature termination of transcription is due to remdesivir incorporation into the nascent viral RNA chains [38]. The antiviral drug remdesivir has been used to treat MERS-CoV, SARS-CoV, and Ebola patients [39], and the drug has shown its efficacy against SARS-CoV-2 recently. The neuraminidase inhibitors peramivir, zanamivir, and oseltamivir were re-evaluated in order to identify potent inhibitors of SARS-CoV-2, since studies do not recommend their use in the treatment of SARS-CoV-2 [40]. Up to date, several anti-inflammatory molecules, such as glucocorticoids, chloroquine and hydroxychloroquine, peptides, immunosuppressants, and cytokine inhibitors (namely IL-6R monoclonal antagonists, TNF-inhibitors, and IL-1 antagonists) have been repurposed to investigate their potential in the treatment of COVID-19 patients.

The anti-malarial and autoimmune drug chloroquine has recently been identified as an effective antiviral drug [41,42]. In vitro studies with Vero E6 cells show that the chloroquine mechanism works by modulating endosomal pH to prevent SARS-CoV-2 invasion during the entry and post-entry stages [43,44]. Chloroquine displays an inhibitory potential against SARS-CoV-2 which has recently been reassessed on an open label and may also be responsible for the modulation of host-pathogen interactions. A few other clinical studies have reported the overall efficacy of glucocorticoids in the treatment of pneumonia in patients with SARS-CoV and MERS-CoV. In order to minimize manifestations raised due to elevated cytokine levels, patients with SARS-CoV-2 were typically prescribed glucocorticoids to prevent inevitable complications [45]. A recent study elaborated on glucocorticoid therapy, in which glucocorticoids were given for a short duration to treat ARDS. For COVID-19 patients, randomized clinical trials have been conducted; however, the therapeutic efficacy of glucocorticoids is still not endorsed. Therefore, glucocorticoid therapy was found invalid and incompetent. Overall, the prognosis and existing evidence suggest that the recovery of COVID-19 patients through glucocorticoid administration is poor, and the application of such therapy may have adverse effects [46]. However, glucocorticoids combined with thalidomide have been used to determine whether the combinatorial approach is effective against SARS-CoV-2 infections. Moreover, a thalidomide and glucocorticoid combination was reported to reduce the hyperinflammatory surge and gastrointestinal symptoms with fewer side effects in COVID-19 patients [47].

Recently, some of the anti-inflammatory molecules such as baricitinib, fedratinib, and ruxolitinib were repurposed and identified to show inhibitory potential against SARS-CoV-2 infections [48]. The same study showed that all three of these drugs effectively inhibit clathrin-mediated endocytosis, thereby reducing elevated levels of cytokines through inhibition of the Janus kinase–signal transducers and activators of transcription (JAK–STAT) signaling pathway in COVID-19 patients. Nitazoxanide is an antiviral drug that has been shown to inhibit SARS-CoV-2, and it effectively potentiates innate immunity and ameliorates the suppressed interferon pathway [49]. The studies documented that some other drugs have potential efficacy in treating SARS-CoV-2, and one of them is darunavir, a protease inhibitor that inhibits viral replication significantly; therefore, the drug might be helpful in the treatment and management of SARS-CoV-2 [50]. Korean scientists recently illustrated that the protease inhibitors lopinavir and ritonavir significantly decrease viral titer after the administration of drugs; however, the authors also indicated the possibility of the natural course of the healing process rather than the administration of lopinavir/ritonavir [51]. Further possibilities in the direction of drug development included several other antiviral drugs that have been investigated, predominantly against various RNA-containing viruses. These drugs may include ritonavir, azvudine, sofosbuvir, favipiravir, triazavirin, and emtricitabine, which have been repurposed to check their efficacy in treating SARS-CoV-2. 

Elevated cytokine levels and other SARS-CoV-2 complications have prompted the research community to focus on immunomodulatory molecules, as hyperinflammation and cytokine storm contribute to the mortality of SARS-CoV-2-infected people. Such re-investigated molecules may include sarilumab (anti-IL-6), adalimumab (anti-TNF), eculizumab (anti-C5), and meplazumab (anti-CD147) [52]. Additionally, CD147, a spike protein, was reported to be acting as a novel receptor for a SARS-CoV-2 invasion in the host cells, and the discloser indicated antiviral activities of meplazumab against SARS-CoV-2, which target CD147 to inhibit virus invasion [53]. Tocilizumab (an IL-6 blocker) was shown in a retrospective study to significantly improve clinical symptoms, reducing the aggravation of SARS-CoV-2, and may be an excellent drug for combating and decreasing SARS-CoV-2 infections [54]. In COVID-19 patients, normal lymphocyte counts and CRP levels were observed following the improvement in body temperature and respiratory functions after tocilizumab administration; therefore, the drug can be effective for targeting the IL-6 signaling cascade [55]. An orally bioavailable prodrug, EIDD-2801, was also demonstrated to show promising therapeutic effectiveness against SARS-CoV-2. In vitro studies indicated improved clinical findings, such as reduced virus titers, healing pulmonary function, and body loss [56]. Another repurposed drug is ivermectin, which may warrant positive outcomes in the development of preventive and therapeutic strategies for treating SARS-CoV-2-infected individuals. In vitro studies (Vero-hSLAM) reported that ivermectin significantly reduced viral RNA to restrict SARS-CoV-2 proliferation, possibly through inhibiting nuclear transport activity, which might be responsible for replication inhibition of the virus [57].

## 4. Mucormycosis and SARS-CoV-2 Comorbidity

Mucormycete is a member of the genera *Mucor rhyzuprhizopusdia* and *Cunninghamella,* and it causes mucormycosis or zygomycosis (black fungus). In recent years, it has emerged as a serious health issue in Asian countries, especially in India. Furthermore, it was reported that COVID-19 patients can also be coinfected with black fungus, which may persist as a potential comorbidity in COVID-19 patients. Black fungal infection is also known as an opportunistic fungal infection, which may alleviate post-SARS-CoV-2 sepsis. Notably, COVID-19 patients have a weakened immune system due to the medications, rendering the patients of SARS-CoV-2 more susceptible to other fetal infections, including mucormycosis [58]. COVID-19 patients are affected by mucormycosis as a result of extended steroid treatment. Elevated iron levels in the serum of SARS-CoV-2 survivors is also the main contributor to the rise in mucormycosis cases. In order to cure mucormycosis, it is necessary to search for potential antimycotic drugs that are more potent and less harmful. The SARS-CoV-2 infection is characterized by a wide spectrum of clinical complications. A recent case report showed that a diabetic patient recovering from SARS-CoV-2 infection was followed by endobronchial necrosis as a result of pulmonary mucormycosis infection, which was confirmed by histopathological examinations and computed tomography [59]. Another study showed that four patients aged more than 42 years were administered with steroids, and half of them had comorbidities of pulmonary mucormycosis and SARS-CoV-2, in which computed tomography revealed pulmonary cavitation and empyema [60]. Patients with underlying comorbidities (diabetes and chronic kidney disease) may have a greater risk of fungal and bacterial infections. Another case series revealed that two chronic kidney disease (CKD) patients became infected with SARS-CoV-2 and mucormycosis while receiving steroids in a long-term intensive care unit [61]. COVID-19 patients were shown to be infected with acute invasive rhino-orbital mucormycosis, which is a highly morbid infection that primarily affects immunosuppressed individuals [62]. In another study, 31 patients with COVID-19-associated rhino-orbito-cerebral mucormycosis (CAROCM) were included, in which a higher incidence of uncontrolled diabetes and renal dysfunction was recorded, while levels of IL-6, ferritin, and D-dimer were significantly higher in CAROCM than in non-mucormycosis patients [63].

## 5. Can Plant Products Overcome the Burden of SARS-CoV-2?

Previous studies have highlighted the importance of plants and their products in the management of infectious diseases, including bacterial, viral, and fungal infections, as well as other diseases such as cancer, malaria, and so on. Therefore, exploring the potential of the plants and their products might be an excellent choice to improve the health of COVID-19 patients, either alone or in combination with other drugs. Medicinal plants are valuable sources of numerous health-beneficial products, as well as having been reported to possess various biological actions. Considering plant products for the prevention and therapeutics of SARS-CoV-2 is essential, as they are praised for their various excellent advantages, including lower toxicity, cost-effectiveness, solubility, and rapid renal clearance. In general, plants are categorized as having terpenes, alkaloids, organosulfur compounds and polyphenols. Furthermore, polyphenols are classified as flavonoids, phenolics, stilbenes, and lignans. 

Polyphenols and alkaloids are the most widespread plant-based products with prominent properties including anti-cancer, antioxidant, antimalarial, antiviral, antibacterial, antifungal, anti-diabetic, anti-inflammatory, and anti-dengue effects. Accordingly, these phytochemicals can be promising candidates for discovering effective therapeutic regimens for SARS-CoV-2. Up to now, numerous studies have shown the potential of phytochemicals against SARS-CoV-2. Molecular insights revealed that phytochemicals and their products can exert anti-SARS-CoV-2 activities through targeting pathogenesis-related proteins of SARS-CoV-2 [64]. In this review, we outline several plant-based products that have been known for their anti-inflammatory and antiviral activities against the coronavirus. We assume that, in addition to their antiviral properties, plant products could be effective in relieving inflammation and disease complications. Overall, the purpose of the review article is to highlight the importance of plant products, so that researchers can consider the plant products for their applicability in the management of SARS-CoV-2 through understanding the underlying mechanisms of actions that might be helpful in overcoming the SARS-CoV-2-associated problems.

### 5.1. Plant Products with Anti-Inflammatory and Antioxidant Potential in Various Diseases

Curcumin has been known for centuries to have anti-inflammatory effects, and extensive studies have shown that curcumin may inhibit inflammation-associated signaling pathways in many pathological conditions [65]. Several studies have shown that IL-6 down-regulation is associated with the therapeutic effects of curcumin [66]. Another study demonstrated that curcumin showed anti-inflammatory effects through the inhibition of TNF-α, serumIL-6, and CRP [67]. Another study elucidated the important role of chloroquine and amodiaquine, which earlier had been implicated in the treatment and prevention of malaria and inflammatory disease (Table 1). Both chloroquine and amodiaquine have been shown to inhibit IFN production [68]. TNF-α, NF-, IL-1, and IL-6 are known key regulators in the pathogenesis of rheumatoid arthritis and other inflammatory diseases, and chloroquine has been shown to inhibit IL-1, IL-6, and TNF- production in human monocytes stimulated with lipopolysaccharide [69]. In contrast, hydroxychloroquine has been shown to inhibit IL-1 and IL-6 production in monocytes while leaving IL-2, IL-4, IFN-, and TNF- production unaffected [70]. In a study, chloroquine and hydroxychloroquine were found to exhibit anti-inflammatory potential against phytohemagglutinin and lipopolysaccharide-stimulated peripheral blood mononuclear cells (PBMC). They discovered that chloroquine and hydroxychloroquine both inhibited IFN- and TNF-production equally [71]. Epigallocatechin gallate (EGCG) attenuates SARS-CoV-2 infection by blocking the interaction of SARS-CoV-2 spike protein receptor-binding domain to human angiotensin-converting enzyme 2 [72].

**Figure 1:**
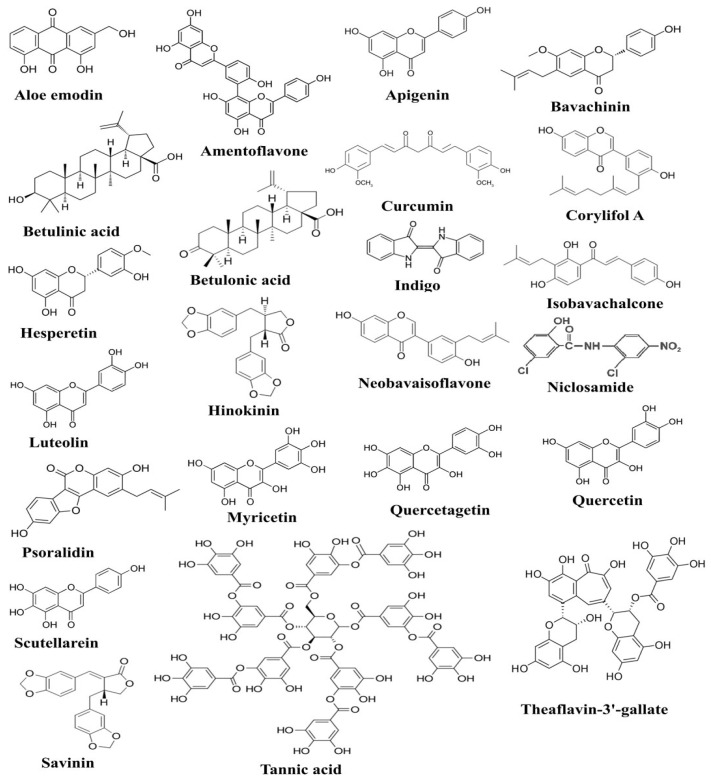
The figure shows chemical structures of plant-derived compounds active against SARS-CoV-2, which have been documented to exert anti-SARS-CoV-2 activities through targeting various pathogenesis-related proteins of SARS-CoV-2 [80].

Anti-inflammatory potential of ascorbic acid has been accomplished by its outstanding power to inhibit the production of IFN-γ while fucoxanthin decreased TNF-α production in human lymphocytes [73]. Ascorbic acid has also been shown to possess immunomodulatory activity, as it inhibits TNF-α and IL-6 production in lipopolysaccharide (LPS)-induced monocytes [74]. Previous studies also indicated the role of ascorbic acid in mitigating the production of TNF-α and IL-6 in LPS-induced macrophages and community-acquired pneumonia [75]. In a few young healthy males, the level of IL-6 was measured before and after the administration of ascorbic acid, and decreased IL-6 production was reported in endothelin-1-induced males [76]. Emodin, an active component of many plants, has previously been reported to prevent the binding of SARS-CoV to ACE2 receptors through the inhibition of spike glycoprotein [77]. Epigallocatechin-3-gallate (EGCG) is a polyphenol found in green tea, and it has been identified with anti-inflammatory, antioxidant, antiviral, and other health-enhancing properties. In vitro studies revealed that polyphenols inhibited the production of many anti-inflammatory cytokines, including IL-6, TNF-α, and IL-8, by inhibiting the cascade that leads to the activation of ERK1/2 and NF-κB [78]. Another study annotated the possible mechanism of EGCG to inhibit Ang II and CRP expression in macrophages [79]. Chemical structures of some of the bioactive compounds active against SARS-CoV-2 and 3C-like proteinase (3CL pro) are presented in Figure 1.

Polyphenols such as punicalagin, gallic acid, and ellagic acid are well-known antioxidants and have been demonstrated previously to inhibit LPS-stimulated IL-6 production [81]. The recent evidence has shown that punicalagin, a component of pomegranate attenuated the production of IL-6 and TNF-α in lipopolysaccharide (LPS)-induced macrophages [82]. Triphala compounds chebulagic acid, chebulinic acid and gallic acid were also reported to display anti-TNF-α activity in addition to the decreased expression levels of IL-6, IL-8 and MCP-1 which is mediated by inhibition of p38, ERK and NFκB phosphorylation [83]. The anti-inflammatory and antioxidant effects of the phenolic compound resveratrol have been known for decades. In vitro studies reported that resveratrol inhibited TNF-α, IL-8 and IL-6 along with the reduced expression of MCP-1 in human coronary artery smooth muscle cells through the attenuation of extracellular signal-regulated kinase (ERK) [84]. The effect of resveratrol on inflammation and oxidative stress in COPD rats showed that the serum levels of IL-6 and IL-8 were significantly decreased upon treatment with resveratrol [85]. Moreover, the flavonoid quercetin was demonstrated to possess anti-inflammatory and antioxidant potential. Quercetin has been shown to reduce IL-6, TNF-α, and IL-1 production in the LPS-induced macrophage cell line RAW264.77 [86].

Luteolin, a plant product naturally present in several medicinal plants, has attracted wide attention for its health benefits. It significantly reduced the production of TNF-α-induced MCP-1, intercellular cell adhesion molecule (ICAM)-1 and vascular cell adhesion molecule (VCAM)-1 through the inhibition of nuclear factor kappa B (NF-κB) signaling [87]. In vivo studies showed that luteolin and curcumin synergistically inhibited protein expression of MCP-1 and VCAM-1 in TNF-α-induced monocytes [88]. Apigenin, a flavonoid found in many plants, possesses various health benefits, and several studies have elucidated its anti-inflammatory and antioxidant properties. Several studies found its anti-inflammatory effects in various diseases, including diabetes, sepsis, cancer, atherosclerosis, and other inflammation-based diseases. A study demonstrated that apigenin potentially reduced the expression of many inflammatory molecules such as TNF-α, IL-6, IL-8, and GM-CSF through modulating the expression of NF-κB [89,90]. Fisetin, a flavone found in many plants, was discovered to inhibit IL-6, TNF-α, MCP-1, and IL-8 production in IL-1β-stimulated human lung epithelial cells by inhibiting NF-κB and ERK1/2 [91]. Rutin, a flavonoid present in several fruits, vegetables and medicinal plants has been reported to alleviate the expression of TNF-α and nitric oxide in LPS-induced macrophages [92]. In vitro studies of 3,4-dihydroxytoluene, a Rutin metabolite, revealed that it inhibited nitric oxide (NO), cyclooxygenase (COX)-2, and inducible nitric oxide synthase (iNOS) production, as well as the expression of inflammatory cytokines such as IL-6, TNF-α, and IL-1 in LPS-stimulated macrophages [93].

Kaempferol, a flavonoid present in several plant species, has been shown to mollify the secretion of MCP-1 in LPS-induced macrophages [94]. Kaempferol7-O-β-D-glucoside is a natural flavonol glucoside. In vitro studies show that it has the potential to reduce TNF-α, IL-6, IL-1β, iNOS, and COX-2 expression by inhibiting the NF-κB, AP-1, and JAK-STAT signaling pathways in LPS-induced macrophages [95]. Myricetin, a member of the flavonoid class of polyphenols, exhibits antioxidant potential, and the polyphenolic compound has been investigated for its anti-inflammatory properties. A recent study illustrated that myricetin might be responsible for reducing the production of IL-6, TNF-α, NO, and iNOS by suppressing the NF-κB and STAT-1 activation and Nrf-2 mediated Heme oxygenase-1(HO-1) induction in LPS-treated macrophages [96]. Hesperetin, a naturally occurring flavonoid in a few plant species, has been investigated for various health-improving benefits in addition to its antioxidant property. The polyphenolic compound inhibited IL-1β, IL-6 and TNF-α production by modulating the expression of peroxisome proliferator-activated receptor (PPAR)-γ and NF-κB in the bronchoalveolar lavage fluid of rats with ventilator-induced acute lung injury [97]. Another in vitro study suggested that hesperetin treatment may suppress IL-6, TNF-α and IL-1β production and iNOS and COX-2 expression which might be through the inhibition of NF-κB and activation of nuclear factor erythroid 2–related factor 2 (Nrf-2)/HO-1 pathways [98].

Naringenin, a polyphenolic flavonoid found in many citrus fruits and herbs, has been shown to reduce acute inflammation in macrophages by inhibiting IL-6 and TNF-αsecretion [99]. The other study looked into its anti-inflammatory properties and discovered that naringenin inhibited the production of IL-6, TNF-α, and IL-1β. Additionally, abated neutrophil and mononuclear cell recruitment and abrogated myeloperoxidase (MPO) activity were shown to reduce LPS-stimulated inflammatory pain, which possibly occurred through inhibition of the NF-κB pathway [100]. Isorhamnetin was also found to inhibit IL-6 production through the inhibition of NF-κB and STAT-1 signaling pathways in LPS-stimulated macrophages [101]. Some of the flavonoids including chrysin, galangin, quercetin, kaempferol and myricetin were also reported to inhibit IL-1β expression in LPS- and IFN-γ-treated macrophages [102]. Theaflavin, a tea polyphenol, has been known for its antioxidant and anti-cancer properties, and the plant product was also investigated for its anti-inflammatory effects. Theaflavin inhibited LPS-stimulated expression of IL-6, MCP-1, and ICAM-1 by modulating the expression of NF-κB and MAPK [103]. Genistein, an isoflavone mainly present in soybeans, exerts beneficial effects. The isoflavones inhibited IL-6 and TNF-α through suppressing NF-κB pathway activation in LPS-stimulated macrophages [104]. However, attenuated iNOS-derived NO expression was demonstrated in LPS-stimulated macrophages [105]. Baicalein treatment significantly reduced STAT1, STAT3, JAK1 and JAK2 phosphorylation in LPS-induced macrophages which alleviated the production of IL-6, IL-1β and TNF-α and NO [106]. Ferulic acid modulated LPS-induced macrophages expression of anti-inflammatory cytokines such as TNF-α, IL-6 and IL-10 by targeting NF-κB and Nrf-2 pathways [107]. Table 1 shows the anti-inflammatory effects of various phytochemicals.

**Table 1:** This table shows various plant products which exert anti-inflammatory potential through targeting various inflammation-related signaling molecules. Several phytochemicals and plant extract have been reported to modulate activities of TNF-α, ILs and IFN-γ. Additionally, they also inhibit the binding of spike protein (ACE2 receptor), cyclooxygenase-2, iNOS and CRP production. Thus, plant extracts and phytochemicals can achieve its anti-inflammatory effects against SARS-CoV-2.

Class	Plant Product	Effects	Reference
Phenolics	Curcumin	Inhibits TNF-α, serum IL-6 and CRP production.	[65,67]
Phenolics	Chloroquine	Inhibits IFN-γ, IL-1, IL-6 and TNF-α production.	[68,69]
Phenolics	Punicalagin	Inhibits IL-6 and TNF-α production.	[81,82]
Phenolics	Ascorbic acid	Inhibits IFN-γ, TNF-α and IL-6 production.	[73,74]
Anthraquinone	Emodin	Inhibits binding of spike protein to ACE2 receptor.	[77]
Flavanol	EGCG	Inhibits IL-6, TNF-α and IL-8 production.	[78]
Phenolics	Hydroxychloroquine	Inhibits IL-1, IL-6 IFN-γ and TNF-α production.	[70,71]
Flavone	Luteolin	Inhibits production of TNF-α-induced MCP-1, ICAM-1 and VCAM-1.	[87]
Flavone	Apigenin	Inhibits TNF-α, IL-6, IL-8, and GM-CSF expression.	[89,90]
Carotenoid	Fucoxanthin	Decreases TNF-α production.	[73]
Phenolics	Resveratrol	Inhibits TNF-α, IL-8 and IL-6 and MCP-1 expression.	[84]
Phenolics	Gallic acid	Inhibits IL-6 production.	[81]
Phenolics	Chebulagic acid (Triphala)	Inhibits IL-6, IL-8 and MCP-1 expression.	[83]
Phenolics	Chebulinic acid (Triphala)	Inhibits IL-6, IL-8 and MCP-1 expression.	[83]
Flavonol	Quercetin	Inhibits IL-6, TNF-α and IL-1β production.	[86]
Flavonol	Fisetin	Inhibits IL-6, TNF-α, MCP-1 and IL-8 production.	[91]
Flavone	Rutin	Inhibits expression of NO and TNF-α.	[92]
Flavonol	Kaempferol	Inhibits secretion of MCP-1.	[94]
Phenolics	Ellagic acid	Inhibits IL-6 production.	[81]
Flavonol	Myricetin	Inhibits IL-6, TNF-α, NO, and iNOS.	[96]
Flavanone	Hesperetin	Inhibits IL-1β, IL-6 and TNF-α production.	[97,98]
Flavanone	Naringenin	Inhibits IL-6 and TNF-α production.	[99]
Glycoside	Isorhamnetin	Inhibits IL-6 production.	[101]
Flavone	Chrysin	Inhibits IL-1β expression.	[102]
Flavonol	Galangin	Inhibits IL-1β expression.	[102]
Phenolics	Theaflavin	Inhibits expression of IL-6, MCP-1 and ICAM-1	[103]
Isoflavonoid	Genistein	Inhibits IL-6 and TNF-α production.	[104]
Flavone	Baicalein	Inhibits production of IL-6, TNF-α, IL-1β and NO.	[106]
Phenolics	Ferulic acid	Inhibits production TNF-α, IL-6 and IL-10	[107]
Flavonol	Kaempferol7-o-β-d-glucoside	Inhibits expression of TNF-α, IL-6, IL-1β, iNOS and COX-2.	[95]

### 5.2. Antiviral Activities of Plant Products: An Insight into Molecular Mechanisms

Various antiviral plant products and their mechanisms of action have been suggested in this section. Several studies have shown the therapeutic potential of many plant products, which include flavonoids, alkaloids, terpenoids, chalcones, glycosides, phenolics, tannins, and lignins. There are many molecular targets that had been anticipated in view of finding a potent target that may lead to the development of a novel strategy for the prevention and treatment of coronaviruses. Various possible molecular targets were identified in order to treat and prevent the previously occurring SARS-CoV and MERS-CoV outbreaks through the identification and application of antiviral plant products. These molecular targets include spike glycoproteins S1 and S2 (domains), virus-encoded proteases such as 3C-like cysteine protease (3CL pro) and papain-like cysteine protease, and virus-encoded enzymes such as RNA-dependent RNA polymerase and NTPase/helicase. Herein, we repurposed several plant products to consider them as the possible hope to controlling the novel pandemic, as earlier many plant products such as chloroquine and hydroxychloroquine have proven their anti-malarial potential (Table 2).

Hesperidin is commonly found in citrus fruits, and its aglycone metabolite is known as hesperetin. In a recent study, hesperetin and hesperedin were considered to determine whether they exhibit inhibitory activities against SARS-CoV-2. The results of the in-silico study showed that both compounds have the ability to bind with TMPRSS2 and ACE2, and they suppressed the infection of VeroE6 cells with SARS-CoV-2 by inhibiting the interaction between S protein and ACE2, which indicates that both compounds can be used for the treatment and prevention of SARS-CoV-2 [108]. Honokiol, the main compound found in Magnolia trees, has previously been shown to have anti-cancer and anti-inflammatory properties. In an interesting study, honokiol was shown to inhibit viral growth and proliferation in cell culture. Notably, honokiol protected VeroE6 cells from the SARS-CoV-2 infection with an EC50 value of 7.8 µM, decreasing the viral RNA copies and viral titers. Further investigations showed that honokiol inhibited SARS-CoV-2 replication, and they also suggested that honokiol is also active against many variants of SARS-CoV-2, including the Omicron variant and other human coronaviruses [109].

In a recent study, *Allium sativum*, *Nigella sativa*, *Camellia sinensis*, *Curcuma longa,* and *Eucalyptus* sp. were analyzed for phytochemicals, which revealed that naringenin and saikosaponins show promising anti-SARS-CoV-2 effects [110]. Another study investigated a library of plant products and identified myricetin and scutellarein as the novel inhibitors of the SARS-CoV helicase, nsP13 [111]. Quercetin and epigallocatechin gallate are some of the important flavonoids which played pivotal roles in improving the health status, as well as fighting against many pathogens. They were investigated for their antiviral potential due to antioxidant and anti-inflammation properties and were found to stop SARS-CoV replication via the amelioration of 3CLpro expressed in *Pichia pastoris* [112]. *Ricinus communis*, a medicinal plant, is known for its various pharmacological properties, including antioxidation, antibacterial, antiviral, and anti-inflammatory actions, which were studied to determine its anti-SARS-CoV-2 effects. The studies showed that *Ricinus communis* containsricinine and lupeol as their chief phytoconstituents, and a methylene chloride extract of this plant exerts prominent virucidal activity against SARS-CoV-2. However, they further showed that ricinine exhibited superior anti-SARS-CoV-2 effects with an IC_50_ value of 2.5 µg/mL, followed by lupeol with an IC_50_ value of 19.5 µg/mL [113].

In an in-silico study, luteolin was reported to bind with the spike glycoprotein, RdRp, and ADP phosphatase of non-structural protein-3, suggesting luteolin as an anti-SARS-CoV-2 compound [114]. Four flavonoids, amentoflavone, luteolin, apigenin, and quercetin, have been demonstrated to inhibit the SARS-CoV; however, amentoflavone and luteolin significantly inhibited 3CL proteinase (3CL pro) activity at lower doses, comparatively [115]. Another study screened several phytochemicals for their potential activities against 3CL pro and PL pro, and out of 32 phytocompounds, only amentoflavone and gallocatechin gallate were shown to possess the best binding with 3CL pro and PL pro, which may help in the development of amentoflavone and gallocatechin gallate as drug candidates against 3CL pro and PL pro as therapeutics for SARS-CoV-2 [116]. An aqueous extract of *Houttuynia cordata* was found to be enriched with flavonoids such as quercetin, quercitrin, and isoquercitrin, which demonstrated the inhibition of herpes simplex virus (HSV) and SARS-CoV [117]. Five *Isatisindigotica* root-extracted compounds and seven plant-derived compounds were evaluated for their anti-SARS-CoV activities, and in vitro studies suggested that hesperetin, sinigrin, and aloe emodin were observed as the most effective inhibitors of cleavage activity of the 3CL pro [118]. Another in silico study has screened 41 antiviral compounds from Indian medicinal plants, and among them, amentoflavone, hypericin, and torvoside H were suggested to serve as potential inhibitors against SARS-CoV-2 [119].

Glycyrrhizin, a compound mainly found in *Glycyrrhiza glabra,* exerts anti-inflammatory activities and has also been investigated for its anti-SARS-CoV effects. One of the research articles has indicated that glycyrrhizin may inhibit the replication of SARS-CoV at higher doses, which may cause toxicity to normal cells as well [120]. Another study demonstrated that glycyrrhizin and licorice extract possess multiple health benefits, including antioxidant, antibacterial, and anti-inflammatory activities. Furthermore, the researchers showed that glycyrrhizin and licorice extract exhibited significant inhibitory activity against SARS-CoV-2, which was due to the inhibition of viral entry through the inhibition of viral interaction with the cell membrane receptor ACE2 [121]. Cinanserin, a compound naturally available in *Houttuynia cordata* plant species, inhibited SARS-CoV replication, most likely through the inhibition of 3CL proteinase activity [122]. Lycorine, a compound extracted from the medicinal herb *Lycoris radiata*, demonstrated anti-SARS-CoV activities [123]. The same research article has explored many medicinal herbs for their effectiveness against the SARS-CoV and reported that out of several, only four Chinese medicinal plants, including *Artemisia annua* L. (ethanolic extract), *Lycoris radiata* (ethanolic extract), *Pyrrosia lingua* (chloroform extract), and *Lindera aggregate (sims) kasterm* (ethanolic extract) inhibited the activity of the SARS-CoV [123]. Emodin, a compound specifically extracted from the genera *Rheum* and *Polygonum,* was found to impede the growth of SARS-CoV through the inhibition of spike proteins and ACE2 interaction [77]. Aqueous extracts of *Rheum officinale* Baill and *Polygonum multiflorum* Thunb (Chinese medicinal herbs) significantly inhibited the host–pathogen interaction through the inhibition of spike proteins and ACE2 interactions [77].

**Figure 2:**
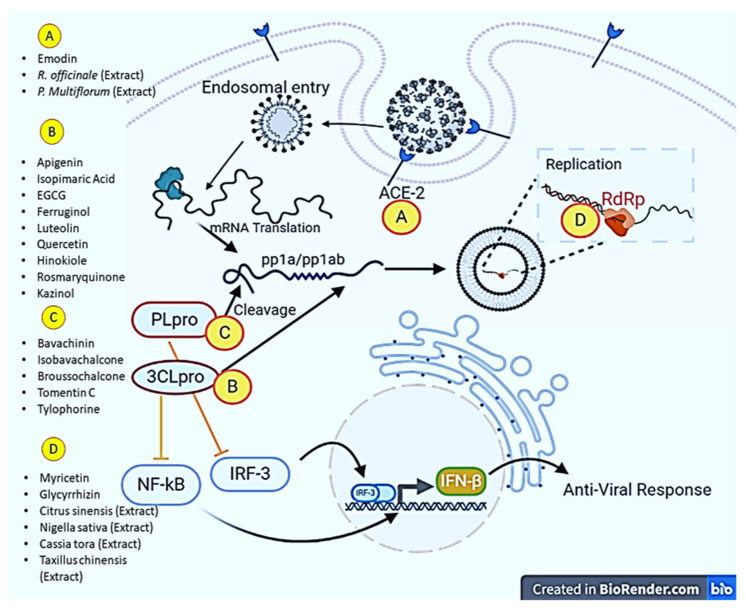
Major molecular targets of phytochemicals in combating SARS-CoV-2. Phytochemicals and plant extracts can target angiotensin-converting enzyme (ACE-2), RNA dependent RNA polymerase (RdRp), Papain-like protease (PL pro) and 3-chymotrypsin-like cysteine protease (3CL pro). The illustration was created at BioRender.com (an online available tool for illustrations).

*Zingiber officinale*, *Scutellariabaicalensis*, *Nigella sativa*, *Allium sativum*, *Camellia sinensis*, *Hypericum perforatum*, and *Glycyrrhiza glabra* were studied to identify phytochemicals and their anti-SARS-CoV-2 activities. The results of the study showed that they possess different types of terpenoids, out of which emodin and baicalin inhibited S protein, iguesterin inhibited 3CL pro, cryptotanshinone targeted PL pro and sotetsuflavone inhibited RdRp, thus they exhibited potent anti-SARS-CoV-2 activities [124]. *Houttuynia cordata* Thunb, a member of the *Saururaceae* family, was screened to check whether the plant displays anti-SARS-CoV activities. As a result, an aqueous extract of this plant inhibited both 3CL pro and RdRp of SARS-CoV [125]. In order to identify the potential inhibitory effects against SARS-CoV, more than 200 Chinese medicinal herbs were screened, but only a few of them showed antiviral activities. *Gentiana scabra*, *Dioscorea batatas*, *Cassia tora*, *Taxillus chinensis*, and *Cibotium barometz* inhibited Vero E6 cell proliferation and viral replication; however, *Cibotium barometz* and *Dioscorea batatas* were found to be significant inhibitors of 3CL pro [126]. *Rheum palmatum*, a Chinese medicinal herb, was also investigated and was found to prevent the replication of SARS-CoV by inhibiting 3CL pro [127]. *Toona sinensis* Roem, a plant species in the *Meliaceae* family, was studied for antiviral effects, and the results showed that the plant inhibited SARS-CoV replication in a promising way [128]. *Prunella vulgaris* and *Saussurea lappa* were studied among 121 Chinese herbs, and the two compounds tetra-o-galloyl-D-glucose and luteolin extracted from these plants demonstrated antiviral potential by inhibiting spike protein S2 and preventing SARS-CoV entry into Vero E6 cells [129]. *Anthemis hyaline, Nigella sativa*, and *Citrus sinensis* extracts have been proven to inhibit the replication of the coronavirus, but the molecular mechanism of the inhibiting replication machinery was not clearly understood [130]. Moreover, recently, two important phytochemicals, curcumin and catechin, were studied for their inhibitory activities against SARS-CoV-2. The results of the in-silico study revealed a high potential to bind with S protein and ACE2, implying that both polyphenolic compounds could be used as anti-SARS-CoV-2 agents [131]. In addition to immunomodulatory, antioxidant, and anti-inflammatory actions, oxidized EGCG also displayed inhibitory activity against SARS-CoV-2 [132]. Furthermore, the flavonoids EGCG and theaflavin were shown to block the virus particle from attaching to the ACE2 and glucose-regulated protein (GRP) 78 receptors. In particular, an in-silico approach revealed that EGCG binds to 3CL pro and both possessed anti-inflammatory properties to control the actions of inflammatory cytokines. All of these experimental results suggest that both flavonoids could be used in the treatment and prevention of SARS-CoV-2 [133]. The impact of bioactive metabolites derived from medicinal plants on molecular targets of various steps of the multiplication process of SARS-CoV-2 has been presented in Figure 2.

Recently, an in-silico study revealed that flavonoids such as apigenin, quercetin, and kaempferol bind with the spike receptor protein of SARS-CoV-2; however, quercetin was found to be the most active phytochemical against SARS-CoV-2 and could be a better inhibitor in combating SARS-CoV-2 infections [134]. In silico and in vitro studies suggested that out of many studied phytochemicals, curcumin, EGCG, theaflavin and resveratrol were found to be effective in impeding the coronavirus [135]. Resveratrol, a polyphenolic compound, has been investigated for its antiviral activities against SARS-CoV-2. The results of the study showed that this compound exhibited anti-inflammatory potential and inhibited SARS-CoV-2 replication in human primary bronchial epithelial cell cultures. The low bioavailability (oral administration) of this compound limits its use in clinical practices; however, a topical administration through inhaled formulations may provide a sufficient amount of resveratrol in the lung airways (the site for the entry of SARS-CoV-2) [136]. In an interesting study, black garlic and 49 polyphenolic compounds were screened for their anti-SARS-CoV-2 activities. The results of the study showed that the black garlic extract exerted its inhibitory effect on purified main protease enzyme (Mpro) with an IC_50_ value of 137 μg/mL, whereas the mixture of tannic acid, daidzein, and puerarin and/or myricetin was found to enhance the inhibitory effects on Mpro [137]. A recent study found that naringenin can be a promising novel pharmacological compound with safe and efficient therapy for SARS-CoV-2 infections, as naringenin strongly inhibited SARS-CoV-2 replication in VeroE6 cells through targeting endo-lysosomal two-pore channels [138].

Some of the biflavonoids and diterpenoids extracted from a traditionally used medicinal plant, *Torreya nucifera*, were predicted to inhibit the 3CL pro of SARS-CoV. The diterpenoids, ferruginol, *o*-acetyl-18-hydroxyferruginol, kayadiol and 18-oxoferruginol also demonstrated inhibitory potential against 3CL pro activity of SAS-CoV, slightly more than other isolated diterpenoids such as methyl dehydroabietate, 18-hydroxyferruginol, and hinokiol andisopimaric acid [115]. The geranylated flavonoids extracted from *Paulownia tomentosa* were found capable of targeting the SARS-CoV papain-like protease (PL pro) in which the compounds tomentin E, tomentin B, and tomentin A were demonstrated as potent inhibitors more comparatively than tomentin C and tomentin D [139]. Many polyphenolic compounds were isolated from the plant species *Broussonetia papyrifera* for the evaluation of anti-PL pro and anti-3CL pro activities in coronaviruses. Among all of the evaluated compounds, papyriflavonol A, broussochalcone A, broussochalcone B, and kazinol J inhibited SARS-CoV-PL pro more effectively in contrast to other polyphenols including 4-hydroxyisolonchocarpin, 3′-(3-methylbut-2-enyl)-3′,4,7-trihydroxyflavane, kazinol A, kazinol B, broussoflavan A, and kazinol F. However, all the polyphenols were also effective against SARS-CoV 3-chymotripsin-like protease [140]. The ethanolic seed extracts from the medicinal plant *Psoralea corylifolia* had earlier demonstrated the significant anti-SARS-CoV PL pro potential. The purification process identified many aromatic compounds such as bavachinin, neobavaisoflavone, isobavachalcone, 4′-O-methylbavachalcone, psoralidin and corylifol A, and was reported to be endowed with good PL pro activities. Both the aromatic compounds psoralidin and isobavachalcone displayed potent efficacies in deterioration of the virus replication [141]. Anti-SARS-CoV plant products also comprise of tylophorine compounds purified from *Tylophora indica* that inhibit the SARS-CoV papain-like protease to curb the viral load via targeting virus replication [142].

With an extending approach in view of repurposing the plant products, *Salvia miltiorrhiza*-derived tanshinones have been reported as inhibiting the coronaviral cysteine proteases. The plant derived tanshinones include tanshinone IIA, tanshinone IIB, methyl tanshinonate, cryptotanshinone, tanshinone I, dihydrotanshinone I, and rosmariquinone. Dihydrotanshinone I, rosmariquinone, and tanshinone IIB suppressed the viral replication by inhibiting the 3CL pro while cryptotanshinone, tanshinone IIA, and dihydrotanshinone I inhibited 3PL pro activity specifically [143]. Anti-SARS-CoV effects of many medicinal herbs including Cinnamomi cortex *(Cinnamomum verum)*, Forsythiae Fructus *(Forsythia suspensa)*, Scutellariae Radix (*Scutellaria baicalensis*), Astragali Radix (*Astragalus propinquus*), Bupleuri Radix (*Bupleurum chinensis*) and Glycyrrhizae Radix (*Glycyrrhiza uralensis*) were also envisaged and only a purified compound, procyanidin, and a *Cinnamomum verum* extract were found to be significantly effective in decreasing coronaviral load while the molecular mechanism was not clearly understood [144]. In an in vitro study, plant-synthesized terpenoids and lignoids were investigated against SARS-CoV replication and proliferation in VeroE6 cells in addition to SARS-CoV 3CL pro. Of the hundreds of plants tested for derived compounds, only ferruginol, 8β-hydroxyabieta-9(11),13-dien-12-one, 7β-hydroxydeoxycryptojaponol, 3β-12-diacetoxyabieta-6,8,11,13-tetraene, betulonic acid, and savinin were found to promote exacerbation of SARS-CoV replication [145]. Table 2 shows the antiviral effects of different plant extracts and their bioactive compounds.

**Table 2:** This table shows the effects of different plant extracts and phytochemicals against SARS-CoV. Notably, these plants products and plant extracts can inhibit various proteins such as nonstructural protein 13 (nsp13), Papain-like protease (PL pro), 3-chymotrypsin-like cysteine protease (3CL pro), angiotensin-converting enzyme (ACE-2) and RNA dependent RNA polymerase (RdRp). Thus, these plant products and extracts inhibit viral binding, entry, replication and proteolytic cleavage of PL pro and 3CL pro.

Plant/Plant Products	Effects	References
Myricetin	Inhibits nsP13 (SARS-CoV helicase).	[111]
Scutellarein	Inhibits nsP13 (SARS-CoV helicase).	[111]
Gallocatechin gallate	Inhibits SARS-CoV 3CL pro.	[112]
Luteolin	Inhibits SARS-CoV 3CL pro.	[115]
Hesperetin (*Isatisindigotica*)	Inhibits cleavage activity of the 3CL pro of SARS-CoV.	[118]
Glycyrrhizin (*Glycyrrhiza glabra*)	Inhibits SARS-CoV replication.	[120]
Cinanserin (*Houttuynia cordata*)	Inhibits SARS-CoV 3CL pro.	[122]
Lycorine (*Lycoris radiata*)	Inhibits SARS-CoV activities.	[123]
Quercetin	Inhibits SARS-CoV 3CL pro.	[112]
Isobavachalcone (*Psoralea corylifolia*)	Inhibits SARS-CoV PL pro.	[141]
Quercitrin (*Houttuynia cordata*)	Inhibits SARS-CoV growth.	[117]
Isoquercitrin (*Houttuynia cordata*)	Inhibits SARS-CoV growth.	[117]
Apigenin	Inhibits SARS-CoV 3CL pro.	[115]
Emodin	Inhibits interactions between spike protein and ACE2 receptor protein.	[77]
Sinigrin (*Isatisindigotica*)	Inhibits cleavage activity of the 3CL pro of SARS-CoV.	[118]
Tomentin A (*Paulownia tomentosa*)	Inhibits SARS-CoV papain-like protease.	[139]
*Lycorisradiata* (ethanolic extract)	Inhibits SARS-CoV activities.	[123]
Methyl dehydroabietate	Inhibits SARS-CoV 3CL pro.	[115]
18-hydroxyferruginol	Inhibits SARS-CoV 3CL pro.	[115]
*Anthemis hyaline*	Inhibits SARS-CoV replication.	[130]
Aloe emodin (*Isatisindigotica*)	Inhibits cleavage activity of the 3CL pro of SARS-CoV.	[118]
*Rheum palmatum* L.	Inhibits SARS-CoV 3CL pro.	[127]
*O*-acetyl-18-hydroxyferruginol	Inhibits SARS-CoV 3CL pro.	[115]
Rosmariquinone (*Salvia miltiorrhiza*)	Inhibits SARS-CoV 3CL pro.	[140]
*Prunella vulgaris*	Blocks viral entry.	[129]
Tomentin B (*Paulownia tomentosa*)	Inhibits SARS-CoV papain-like protease.	[139]
*Rheum officinale* Baill	Inhibits interactions between spike protein and ACE2 receptor protein.	[77]
*Houttuynia cordata* Thunb	Inhibits SARS-CoV 3CL pro and RdRp.	[125]
*Cibotium barometz*	Inhibits SARS-CoV 3CL pro.	[126]
Dihydrotanshinone I (*Salvia miltiorrhiza*)	Inhibits SARS-CoV 3CL pro.	[140]
*Toona sinensis* Roem	Inhibits SARS-CoV replication.	[128]
*Citrus sinensis*	Inhibits SARS-CoV replication.	[130]
18-oxoferruginol	Inhibits SARS-CoV 3CL pro.	[115]
Bavachinin (*Psoralea corylifolia*)	Inhibits SARS-CoV PL pro.	[141]
*Polygonum multiflorum* Thunb	Inhibits interactions between spike protein and ACE2 receptor protein.	[77]
Isopimaric acid	Inhibits SARS-CoV 3CL pro.	[115]
*Pyrrosia lingua (*chloroform extract)	Inhibits SARS-CoV activities.	[123]
EGCG	Inhibits SARS-CoV 3CL pro.	[112]
*Nigella sativa*	Inhibits SARS-CoV replication.	[130]
Hinokiol	Inhibits SARS-CoV 3CL pro.	[115]
Tomentin C (*Paulownia tomentosa*)	Inhibits SARS-CoV papain-like protease.	[139]
Amentoflavone	Inhibits SARS-CoV 3CL pro.	[115]
*Artemisia annua* L. (ethanolic extract)	Inhibits SARS-CoV activities.	[123]
*Gentiana scabra*	Inhibits viral replication and proliferation in Vero E6 cells.	[126]
*Saussurealappa*	Blocks viral entry.	[129]
Tomentin D (*Paulownia tomentosa*)	Inhibits SARS-CoV papain-like protease.	[139]
Psoralidin (*Psoralea corylifolia*)	Inhibits SARS-CoV PL pro.	[141]
Corylifol A (*Psoralea corylifolia*)	Inhibits SARS-CoV PL pro.	[141]
4′-o-methylbavachalcone (*Psoralea corylifolia*)	Inhibits SARS-CoV PL pro.	[141]
Tomentin E (*Paulownia tomentosa*)	Inhibits SARS-CoV papain-like protease.	[139]
*Cassia tora*	Inhibits viral replication and proliferation in Vero E6 cells.	[126]
Papyriflavonol A (*Broussonetiapapyrifera*)	Inhibits SARS-CoV PL pro and 3CL pro.	[140]
Broussochalcone A (*Broussonetiapapyrifera*)	Inhibits SARS-CoV PL pro and 3CL pro.	[140]
Kayadiol	Inhibits SARS-CoV 3CL pro.	[115]
KazinolJ (*Broussonetiapapyrifera*)	Inhibits SARS-CoV PL pro and 3CL pro.	[140]
Tanshinone IIB (*Salvia miltiorrhiza*)	Inhibits SARS-CoV 3CL pro.	[140]
Neobavaisoflavone (*Psoralea corylifolia*)	Inhibits SARS-CoV PL pro.	[141]
*Dioscorea batatas*	Inhibits SARS-CoV 3CL pro.	[126]
Ferruginol	Inhibits SARS-CoV 3CL pro.	[115]
Broussochalcone B (*Broussonetiapapyrifera*)	Inhibits SARS-CoV PL pro and 3CL pro.	[140]
Tylophorine (*Tylophora indica*)	Inhibits SARS-CoV PL pro.	[142]
*Taxillus chinensis*	Inhibits viral replication and proliferation in Vero E6 cells.	[126]
Cryptotanshinone (*Salvia miltiorrhiza*)	Inhibits SARS-CoV PL pro.	[140]

## 6. Conclusions

Cytokine storm, and other associated issues, has been linked to disease severity, and there are currently no approved therapeutics or chemopreventive agents. As of now, therapies being explored include current flu medication, anti-malarial drugs, unsuccessful Ebola drugs, as well as SARS-CoV and MERS-CoV, which were originally developed decades ago. The fast spread of the coronavirus has also encouraged scientists worldwide to investigate the development of vaccines, in addition to the efforts made to produce any potential SARS-CoV-2 medications. A variety of substances, including monoclonal antibodies, peptides, interferons, oligonucleotides, and plant products, have been tested to find potential chemopreventive and chemotherapeutic drug candidates for the prevention and treatment of infectious coronavirus. Plant products have been found to inhibit molecular targets such as myricetin and scutellarein, which inhibit the SARS-CoV helicase. Gallocatechin gallate, quercetin, and epigallocatechin gallate are some of the important flavonoids with antioxidant and anti-inflammation properties that have been investigated for their antiviral potential and were found to stop SARS-CoV replication via the amelioration of 3CL pro expressed in Pichia pastoris. Evidence advocates that plant products (Hesperetin, Quercetin, Apigenin, and Broussochalcone A) may also be a good choice to fight against the present coronavirus infection. Similarly, this review summarizes various plant products that have shown antiviral, antioxidant, anti-inflammation, anti-cancer and anti-parasitic properties in the management of many diseases. Numerous studies have demonstrated that plant products and plant extracts are capable of inhibiting the SARS-CoV-2 virus. Therefore, such herbal formulations may lead to pre-eminent results in clinical trials, as resveratrol has been under clinical trial. Plant products are the best choice for researchers looking for novel, safe, and efficacious plant products that may serve as potent drug candidates to combat the SASR-CoV2 virus due to their low cost, fewer side effects, and rapid renal clearance. In addition, such formulations and supplements rich in polyphenols and alkaloids can also have positive outcomes, as they can be excellent in reducing mortality, and may aid in improving recovery from SARS-CoV-2 infections. However, it is noteworthy that many available anti-SARS-CoV-2 plant products were suggested at a theoretical level because most of the researchers speculated them to be anti-SARS-CoV-2 agents, based on the results of in silico studies. Hence, in vitro and in vivo studies must be conducted to identify their real potential against the SARS-CoV-2 virus. Poor bioavailability, inappropriate pharmacological doses, and low solubility of plant products and extracts are the major limitations that must be resolved before their clinical use against the SARS-CoV-2 virus. Up to now, various variants of SARS-CoV-2 have been reported in different countries, and it will take time to find therapeutic options against each variant. However, different vaccines have been developed for each type of variant, as shown in Table 3.

## Figures and Tables

**Table 3:** Till now, various variants of SARS-CoV-2 have been reported in different countries. Therefore, different vaccines have been developed for the variants of SARS-CoV-2 worldwide.

SARS-CoV-2 Variant	Vaccines	References
B.1.1.7	BNT162b2, mRNA-1273, BBV152/COVAXIN	[146,147,148]
B.1.351	BNT162b2, mRNA-1273, NVX-CoV2373	[146,147,149]
P.1	Sputnik V, CoronaVac, ChAdOx1 nCoV-19 (AZD1222)	[150,151,152]
B.1.617.2	Sputnik V, BNT162b2, ChAdOx1 nCoV-19	[150,153]
B.1.427/B.1.429	mRNA-1273	[154]
P.2	ChAdOx1 nCoV-19 (AZD1222)	[152]
B.1.525	BNT162b2	[155]
B.1.617.3	Sputnik V	[150]
C.37	Gam-COVID-Vac, ChAdOx1-S, Ad5-nCorV, BBIBP-CorV and mRNA-127	[156]
B.1.1.28	ChAdOx1 nCoV-19 (AZD1222)	[152]
BA.4 and BA.5	BNT162b2	[157]
501Y.V2	BBIBP-CorV, ZF2001	[158]

## Data Availability

Available in the manuscript.

## Abbreviations

ACE2: Angiotensin Converting Enzyme-2; SARS-CoV, Severe Acute Respiratory Syndrome Coronavirus; ARDS, Acute Respiratory Distress Syndrome; CRP, C-Reactive Protein; 3CL pro, 3C-Like cysteine protease; Mpro, main protease; PL pro, papain-like protease; RdRp, RNA-dependent RNA polymerase; MERS, Middle East Respiratory Syndrome; PBMC, Peripheral Blood Mononuclear Cells; PCR, Polymerase Chain Reaction; IL, Interleukin; FGF2, Fibroblast Growth Factor-2; GCSF, Granulocyte Colony Stimulating Factor; GMCSF, Granulocyte-Macrophage Colony Stimulating Factor; IFN-γ, Interferon-γ; IP-10, Interferon gamma-induced protein; MCP-1, Monocyte Chemoattractant Protein-1; MIP, Macrophage Inflammatory Protein; NK cells, Natural Killer cells; CD8, Cluster of Differentiation 8; NKG2A, Natural Killer Group 2A; COPD, Chronic Obstructive Pulmonary Disease; ICU, Intensive Care Unit; FDA, Food and Drug Administration; TMPRSS2, Transmembrane Protease Serine 2; LPS, Lipopolysaccharide; EGCG, Epigallocatechin-3-gallate; ERK, Extracellular Signal Regulated Kinase; NFκB, Nuclear Factor kappa B; ICAM, Intercellular Adhesion Molecule; VCAM, Vascular Cell Adhesion Molecule; COX-2, Cyclooxygenase-2;iNOS, Inducible Nitric Oxide Synthase; JAK, Janus Kinase; STAT-1,Signal Transducer and Activator of Transcription-1; Nrf-2, Nuclear factor erythroid 2-related factor-2; HO-1,Heme Oxygenase-1; PPAR, Peroxisome Proliferator Activated Receptor; MPO, Myeloperoxidase; NTPase, Nucleoside Triphosphatase; HSV, Herpes Simplex Virus; N, Nucleocapsid; S, M, E; spike, membrane, envelope proteins; pp1a, pp1b, nonfunctional polypeptides; nsp, nonstructural proteins; RNA (+), positive-sense RNA; and ER, endoplasmic reticulum.
